# Comparative effect of ALA derivatives on protoporphyrin IX production in human and rat skin organ cultures

**DOI:** 10.1038/sj.bjc.6690556

**Published:** 1999-07

**Authors:** A Casas, A M del C Batlle, A R Butler, D Robertson, E H Brown, A MacRobert, P A Riley

**Affiliations:** CIPYP, CONICET and University of Buenos Aires, Argentina; School of Chemistry, University of St Andrews, St Andrews, Fife, UK; National Medical Laser Centre and Department of Molecular Pathology, Royal Free and University College London Medical School, 46 Cleveland Street, London W1P 6DB, UK

**Keywords:** ALA, PDT, ALA derivatives, ALA esters, iron chelators, CP94

## Abstract

Samples of human and rat skin in short-term organ culture exposed to ALA or a range of hydrophobic derivatives were examined for their effect on the accumulation of protoporphyrin IX (PpIX) measured using fluorescence spectroscopy. With the exception of carbobenzoyloxy-D-phenylalanyl-5-ALA-ethyl ester the data presented indicate that, in normal tissues, ALA derivatives generate protoporphyrin IX more slowly than ALA, suggesting that they are less rapidly taken up and/or converted to free ALA. However, the resultant depot effect may lead to the enhanced accumulation of porphyrin over long exposure periods, particularly in the case of ALA-methyl ester or ALA-hexyl ester, depending on the applied concentration and the exposed tissue. Addition of the iron chelator, CP94, greatly increased PpIX accumulation in human skin exposed to ALA, ALA-methyl ester and ALA-hexyl ester. The effect in rat skin was less marked. © 1999 Cancer Research Campaign


*British Journal of Cancer* (1999) **80**(10), 1525–1532
© 1999 Cancer Research Campaign
Article no. bjoc.1999.0556

### Comparative effect of ALA derivatives on protoporphyrin IX production in human and rat skin organ cultures

**A Casas[1], AM del C Batlle[1], AR Butler[2], D Robertson[2], EH Brown[2], A MacRobert[3] and PA Riley[4]**

[1]CIPYP, CONICET and University of Buenos Aires, Argentina; [2]School of Chemistry, University of St Andrews, St Andrews, Fife, UK; [3]National Medical Laser Centre and [4]Department of Molecular Pathology, Royal Free and University College London Medical School, 46 Cleveland Street, London W1P 6DB, UK

**Summary** Samples of human and rat skin in short-term organ culture exposed to ALA or a range of hydrophobic derivatives were examined for their effect on the accumulation of protoporphyrin IX (PpIX) measured using fluorescence spectroscopy. With the exception of carbobenzoyloxy-D-phenylalanyl-5-ALA-ethyl ester the data presented indicate that, in normal tissues, ALA derivatives generate protoporphyrin IX more slowly than ALA, suggesting that they are less rapidly taken up and/or converted to free ALA. However, the resultant depot effect may lead to the enhanced accumulation of porphyrin over long exposure periods, particularly in the case of ALA-methyl ester or ALA-hexyl ester, depending on the applied concentration and the exposed tissue. Addition of the iron chelator, CP94, greatly increased PpIX accumulation in human skin exposed to ALA, ALA-methyl ester and ALA-hexyl ester. The effect in rat skin was less marked.

**Keywords:** ALA; PDT; ALA derivatives; ALA esters; iron chelators; CP94

Photodynamic therapy (PDT) is a cancer treatment involving the irradiation of tissues that have been sensitized by a photosensitizing agent. A recent approach to PDT involves the use of 5-aminolaevulinic acid (ALA) to induce the endogenous synthesis of protoporphyrin IX (PpIX); its clinical use has been recently reviewed by Peng et al (1997).

The extent to which ALA-induced photosensitization is selective depends on the rates of synthesis and metabolism of PpIX (Fukuda et al, 1992). ALA-induced PpIX accumulation has been shown in some cases to be preferentially greater in tumour cells primarily due to the reduced activity of ferrochelatase, the enzyme catalysing the incorporation of ferrous iron (Van Hillegersberg et al, 1992), and a relative enhancement of deaminase activity (Navone et al, 1990).

In vitro evidence suggests that the enzymes of the haem synthetic pathway are constitutively expressed with the exception of ALA synthase, which regulates porphyrin production by modifying the intracellular availability of ALA (Washbrook et al, 1997). Exposure of cells to an external source of ALA results in enhanced porphyrin synthesis and the accumulation of PpIX. This accumulation of porphyrin is increased by inhibition of ferrochelatase, which can be brought about by the use of iron chelators (Berg et al, 1996).

Some iron chelators such as desferrioxamine (Ortel et al, 1993), EDTA (Hanania et al, 1992) and 2,2′-dipyridyl (Walter et al, 1997) have been investigated in order to enhance the quantity of PpIX produced from ALA application with some good results. Orthophenantholine has also been employed (Rebeiz et al, 1992).

It has been previously found that the hydroxypyridinone iron chelator, 1,2-diethyl-3-hydroxypyridin-4-one (CP94), enhanced porphyrin fluorescence and photosensitivity in cell lines (Bech et al, 1997), as well as doubling the PpIX content in the urothelium of normal bladder (Chang et al, 1997) and normal colon mucosa of rats, inducing an increased area of necrosis after PDT (Curnow et al, 1998).

*Received 9 October 1998*
*Revised 4 February 1999*
*Accepted 11 February 1999*

*Correspondence to:* PA Riley

Uptake of ALA from extracellular sources is an important determinant of PpIX accumulation. The mechanism of cellular uptake is not clear in eukaryotic cells, but some transporter mechanism is inferred (Washbrook et al, 1997; Correa Garcia et al, 1998). Entry by diffusion may be limited by the hydrophilic nature of ALA. Therefore, derivatives rendered more hydrophobic by the addition of lipophilic moieties which can be cleaved by intracellular enzymes have been investigated as pro-drugs.

Some lipophilic molecules, such as esterified ALA derivatives, have been used both in human basal cell carcinomas (Peng et al, 1995), in nude mouse skin (Peng et al, 1996) and in human tumour cell lines (Gaullier et al, 1997), and a higher and more homogeneous tissue distribution was produced, compared to those of free ALA-induced porphyrins. In addition, Kloek et al (1996, 1998) demonstrated both in cell lines and in animal models that several ALA pro-drugs are capable of being taken up, de-esterified and converted into PpIX with higher efficiency than ALA itself. However, Washbrook and Riley (1997) and Gaullier et al (1997) found in vitro that ALA-methyl ester (ALA-Me) is less effective than ALA at inducing the synthesis of PpIX.

The aim of this paper is to evaluate by ex-vivo fluorescence, employing skin explants, the capacity of the chelator CP94 and several esterified ALA derivatives of enhancing PpIX synthesis.

### MATERIALS AND METHODS

### Chemicals

ALA powder, ALA-Me (Sigma Chemical Co.) and ALA-hexyl ester (ALA-He) were dissolved in saline. All other ALA derivatives were dissolved in dimethyl sulphoxide (DMSO). The iron chelator CP94 was synthesized according to the procedure of

Hider et al (1982) and was kindly donated by the Department of Pharmacy. King's College, London and used in a concentration of 100 μg $ml^{-1}$ (equivalent to 0.6 mM).

### Synthesis of ALA derivatives

Novel ALA derivatives are depicted in Figure 1, and were synthesized as follows.

ALA-He was prepared according to the method of Takeya (1992) by reacting ALA with hexanol in the presence of thionyl chloride giving the compound as the hydrochloride (HCl) salt.

ALA1–4 were synthesized according to the method of Tanaka et al (1993). The benzyl ester of ALA was prepared by reacting ALA with benzyl alcohol in the presence of thionyl chloride. This was then reacted with the appropriate freshly distilled acid chloride (*N*-butanoyl, *N*-pentanoyl, *N*-hexanoyl or *N*-heptanoyl chloride) in pyridine. The benzyl ester was removed by hydrogenation using 10% palladium on activated charcoal as a catalyst.

ALA5–7 were prepared by reacting a Z-protected amino acid with an ester of ALA using *N*-cyclohexyl-3,2-morpholinoethyl carbodiimide metho p-toluene sulphonate in DCM as a coupling agent.

ALA8 was prepared by reacting *N*-phthalimidolaevulinic acid with glucosamine tetraacetate using the same coupling agent as for ALA5–7. *N*-phthalimidolaevulinic acid was prepared using a method adapted from Iida et al (1998). Tetrahydrofurfuryl chloride was reacted with potassium phthalimide for 3 h (cf. the original method in which tetrahydrofurfuryl bromide was reacted for 1 h) giving *N*-tetrahydrofurfuryl phthalimide. *N*-phthalimidolaevulinic acid (ALA11) was obtained by oxidation using sodium metaperiodate and ruthenium (III) chloride n-hydrate in aqueous acetonitrile and 1,1,2-trichloro-1,2,2-trifluoroethane, a safer substitute for carbon tetrachloride. Glucosamine tetraacetate was prepared using the four-step procedure of Bergmann and Zervas (1932). The amine of glucosamine hydrochloride was first protected using anisaldehyde and acetylation was achieved with acetic anhydride in pyridine. The anisole group was removed with HCl in aqueous acetone and the free amine was released from its HCl salt using sodium methoxide.

ALA9 was prepared by the reaction of ALA with formaldehyde in the presence of hydrogen gas and 10% Pd/C catalyst in water.

ALA10 was synthesized using the method of Kloek and Beijersbergen van Henegouwen (1996) using ALA, acetic anhydride in the presence of triethylamine.

ALA11 was prepared as for ALA8.

### Animal and human skin samples

Animal skin samples were obtained from normal, female Wistar rats (120–200 g) 2 months old. The animals were sacrificed by asphyxia, their skin shaved and excised with scissors, the adipose layer removed and kept on ice until used. Fresh white human skin samples mainly from the breast or abdomen were obtained from cosmetic surgical excisions.

### Skin explant cultures

The explant tissue culture system developed by Polo et al (1988) was used. Skin explants of about 70–100 mg were floated in 2.5 ml of serum-free Dulbecco's modified Eagle's medium (DMEM)–F-12 medium without phenol red, containing 50 IU $ml^{-1}$ penicillin and 50 μg $ml^{-1}$ streptomycin. Incubations were performed in the presence of ALA with or without CP94, or ALA derivatives dissolved in DMSO at 37°C in an atmosphere of humidified air with 5% carbon dioxide in the dark. Control explants incubated in the absence of ALA, and in the presence of DMSO and CP94 alone were performed.

### PpIX content

Direct tissue fluorescence of the epidermal surface was measured with a plate fluorescence reader (Perkin-Elmer, UK) connected to a Perkin-Elmer LS 50B fluorescence spectrophotometer using 410 nm excitation and 635 nm emission with slit widths set to 10 nm both on the excitation and emission monochromators, and an internal 530 nm highpass filter used on the emission side; spectral scans were made between 650 and 780 mm to check for the presence of other porphyrins. Intensity calibrations were performed using a standard of rhodamine B embedded in a Perspex disc. It was shown that fluorescence values were proportional to porphyrin concentration, extracted following the procedure detailed below. Appropriate controls for each specimen were included.

### Spectral identification of porphyrin species

Fluorescence emission spectra from chemically extracted porphyrins were recorded using the Perkin-Elmer LS 50B fluorescence spectrophotometer (Perkin-Elmer, UK) using 410 nm excitation. Protoporphyrin, uroporphyrin I and coproporphyrin I methyl esters (Porphyrin Products, Logan, UT, USA) were used as standards.

The tissue samples were homogenized in a 4:1 solution of ethyl acetate–glacial acetic acid. The homogenates were centrifuged for 30 min at 3000 ***g*** and the supernatants extracted with 5% HCl until the aliquots showed no fluorescence. This procedure extracts over 85% of porphyrins in tissue samples. Porphyrins in the medium were extracted with the same ethyl acetate–acetic acid and HCl procedure. Direct emission spectra of tissue and media porphyrins were also recorded using 410 nm excitation.

### CCD fluorescence microscopy

For recording of fluorescence micrographs skin explants were frozen and 10-μm-thick cryosections were prepared, together with adjacent sections for haematoxylin and eosin staining. A technique combining phase contrast microscopy and a slow-scan charge coupled device (CCD) camera (Wright Instruments Ltd, Enfield, London, UK) was used. The fluorescence was excited using an 8 mW helium–neon laser (632.8 nm) and detected between 665 and 710 nm using bandpass and longpass filters as described previously (Bedwell et al, 1992). A false-colour-coded image of the fluorescence intensity was quantified digitally by averaging over-specified areas. The slow scan CCD system permitted highly reproducible signal calibration. Because the cryosections were of variable quality no quantitative analysis was attempted. Sections of material incubated with ALA, ALA-Me and ALA-He, with and without CP94 were examined.

*© 1999 Cancer Research Campaign*

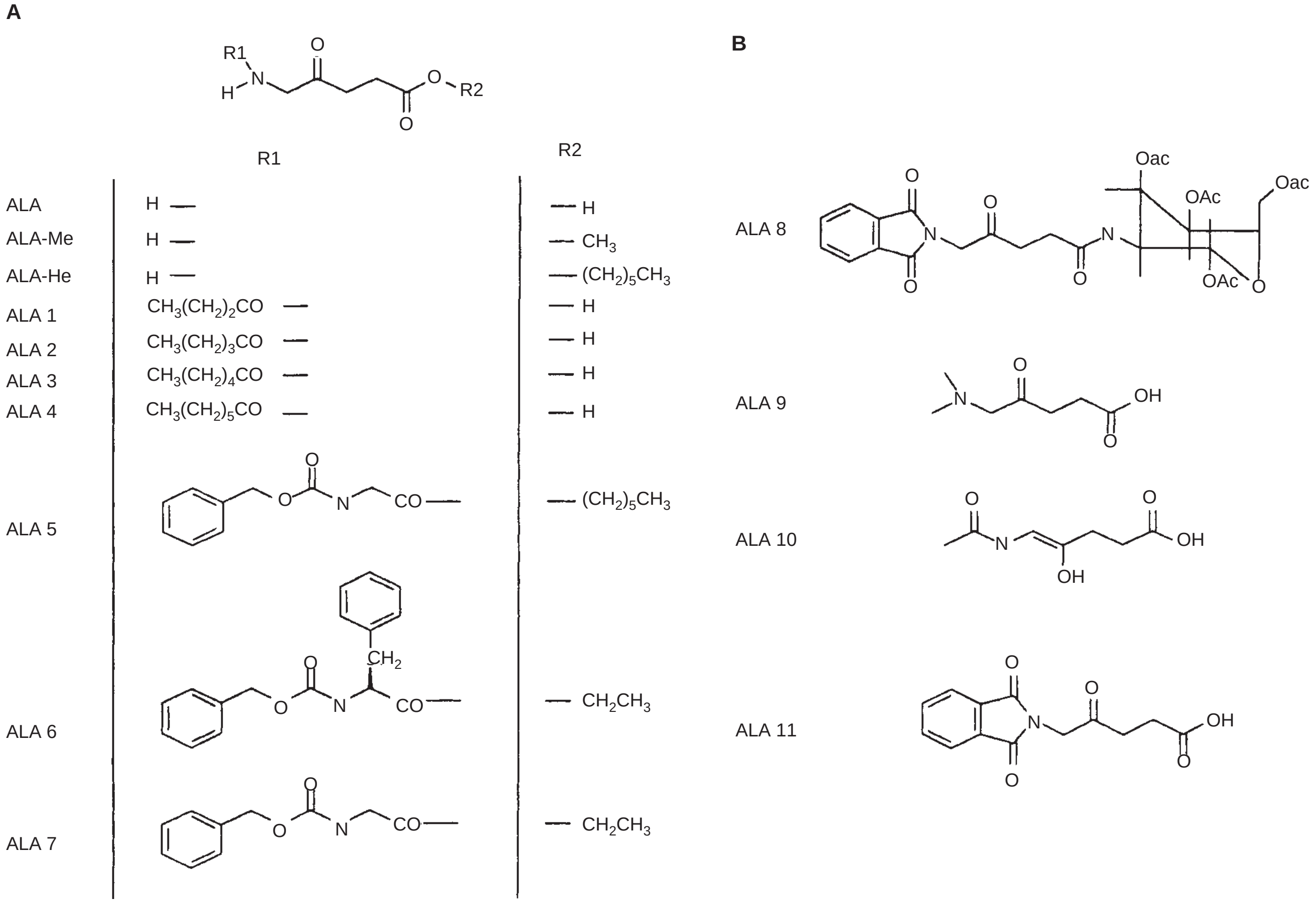

**Figure 1** Structures of ALA and the derivatives. 1: *N*-butanoyl-5-ALA; 2: *N*-pentyl-5-ALA; 3: *N*-hexanoyl-5-ALA; 4: *N*-heptanoyl-5-ALA; 5: carbobenzoyloxy-glycinyl-5-ALA-hexyl ester; 6: carbobenzoyloxy-D-phenylalanyl-5-ALA-ethyl ester; 7: carbobenzoyloxy-glycinyl-5-ALA-ethyl ester; 8: *N*-phthalylimido-5-ALA-glucosamine-tetraacetate; 9: *N,N*-dimethyl-5-ALA; 10: *N*-acetyl-5-ALA and 11: *N*-phthalylimido-5-ALA. Compounds 1, 2, 3, 4, 9 and 10 were obtained as the corresponding hydrochloric acid salts

### Statistical calculation

Statistical analysis of data was performed by Student's *t*-test. Data are reported as mean ± s.d. Changes were considered significant when $P < 0.05$.

### RESULTS

### Spectral identification of porphyrin species

Fluorescence emission spectra of porphyrins extracted from rat skin incubated in the presence of ALA exhibited peaks at 606 and 661 nm coinciding with those of the PpIX standard (Figure 2A).

Coproporphyrin and uroporphyrin were excreted into the medium (Figure 2B) both by human and rat explants exposed to either ALA or its derivatives. The percentage of porphyrins released into the medium was 20% of the total porphyrin synthesized between 2 and 19 h of incubation.

The direct fluorescence emission spectra of explants exposed to ALA (Figure 2C) were characteristic of PpIX with peak emission at 635 and 710 nm as previously demonstrated (Iinuma et al, 1994).

The direct fluorescence spectrum of medium (Figure 2D) showed two prominent peaks at 581 and 616 nm, one of which has been ascribed to hydrophobic porphyrins by Dietel et al (1996), but we have not identified the products.

Medium incubated in the presence of ALA without tissue behaved as control explants incubated without ALA, and no porphyrin peaks were found.

In both rat and human skin explants incubated with ALA-Me, ALA-He and ALA plus CP94, direct or extracted porphyrins showed similar emission spectra to those illustrated in Figure 2A and C, although with differing fluorescence intensities. Similarly, their corresponding medium profiles were similar to those shown in Figure 2B and D.

### PpIX production in skin exposed to ALA, ALA-He and ALA-Me

As Figure 3 illustrates, the levels of rat tissue PpIX after exposure to 0.6 mM ALA increased linearly with time over the 19-h incubation period. Conversely, exposure to either methyl or hexyl derivatives led to a maximum on PpIX synthesis after 5 h of incubation. Nevertheless, 19 h was the time selected for the analysis of concentration-dependence, because human skin exhibited low fluorescence levels at shorter incubation periods (data not shown).

Figure 4A shows that both ALA and ALA-He in rat skin induce maximal PpIX accumulation between 0.6 and 1.2 mM, whereas

© 1999 Cancer Research Campaign

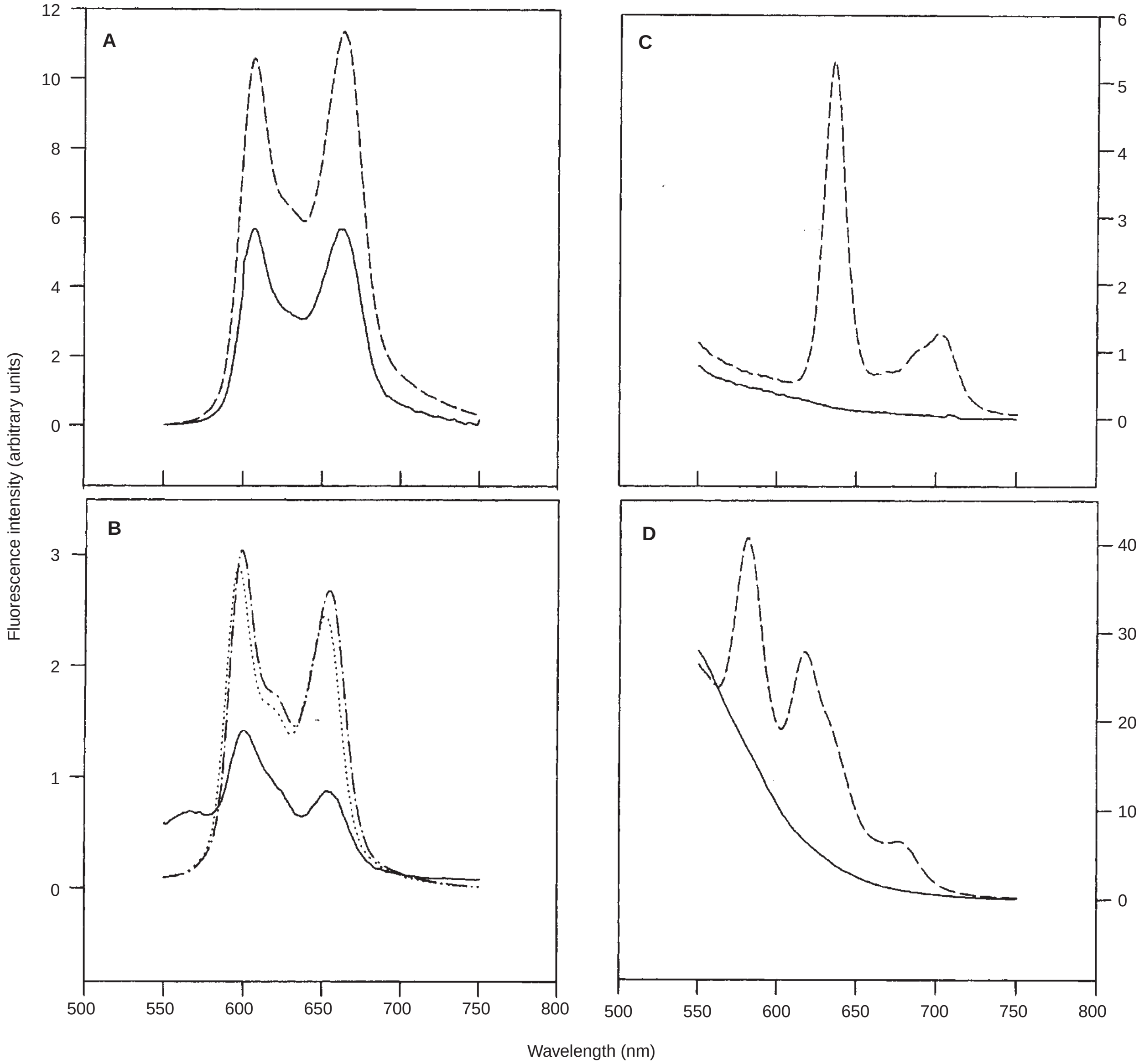

**Figure 2** Fluorescence emission spectra of porphyrins in rat skin and medium. Left panels: fluorescence spectra of skin (**A**) and medium (**B**) after chemical extraction. Solid lines, explant incubated in the presence of 0.6 mM ALA; dashed line, PpIX standard in HCl; dotted line, Coproporphyrin standard in HCl; dash-dotted line, Uroporphyrin standard in HCl. Right panels: direct emission spectra of skin (**C**) and medium (**D**). Dashed lines, explant incubated in the presence of 0.6 mM ALA for 5 h; solid lines, explant incubated for 5 h without ALA

maximal PpIX fluorescence was reached on incubation of tissue with 2 mM ALA-Me.

Figure 4B shows that in human skin explants, synthesis of PpIX is not saturated by ALA or its derivatives within the range of concentrations tested. In both rat and human skin explants ALA is most effective at inducing PpIX fluorescence at low concentrations of substrate (0.2 and 0.6 mM respectively), but the methyl ester appears to have a greater inducing effect ($P$ = 0.01 at higher concentrations – 2 mM). The hexyl ester sample gave significantly lower values than ALA ($P$ = 0.001) at this concentration. Since time courses were not determined for each of the derivatives tested it may be that some of these differences reflect differences in the pattern of uptake as well as relative rates.

### PpIX production by ALA derivatives

In the study of the methyl- and hexyl-ALA effects on PpIX synthesis a number of other ALA derivatives were examined (see Figure 1). Compounds 1, 2, 8 and 11 failed totally to induce PpIX fluorescence and in the case of 1 and 2 they appeared to have toxic effects resulting in lower values than in control skin. The effects of the remaining compounds on rat and human skin explants at three concentrations are summarized in Table 1.

In general, the degree of PpIX induction was less than the corresponding effect of ALA with the exception of compound 6, which exhibited a marginal increase in rat skin ($P$ = 0.001 at 0.6 mM concentration) and levels similar to ALA controls in human skin and slightly higher at 2 mM concentration ($P$ = 0.04).

 © 1999 Cancer Research Campaign

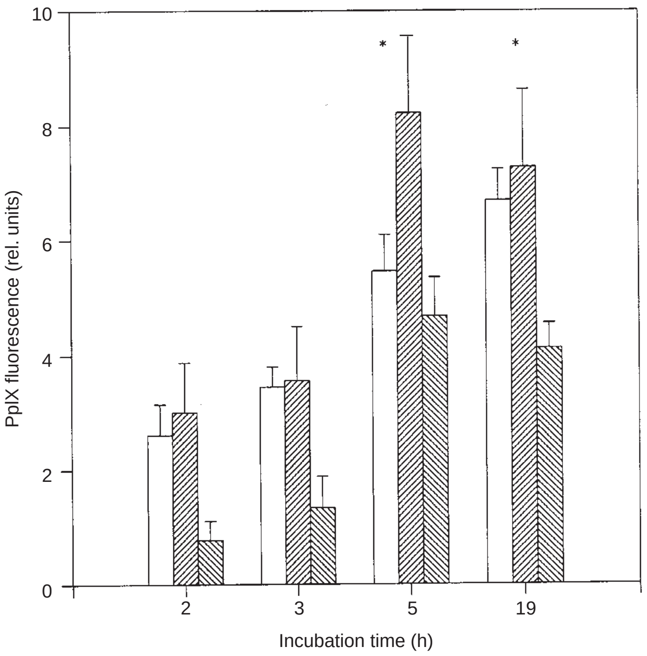

**Figure 3** PpIX fluorescence after exposure of rat skin explants to 0.6 mM ALA (□), ALA-He (▨) and ALA-Me (▧) for 2, 3, 5 and 19 h. Direct skin fluorescence was measured at 410 nm excitation and 635 nm emission, and autofluorescence of explants incubated without ALA was subtracted. The bars show the means of four independent experiments performed in duplicate. Error bars show standard deviations. Asterisks denote $P < 0.05$.

### CP94 enhancement of PpIX production

Figure 5A shows that the simultaneous exposure of rat skin explants to 0.6 mM ALA and CP94 results in a slight increase in PpIX fluorescence ($P = 0.016$ at 200 µg ml$^{-1}$) but in human skin explants (Figure 5B) exposure to CP94 substantially increases the PpIX yield reaching a maximum of about eightfold at 300 µg ml$^{-1}$. This effect was also observed at higher ALA concentrations although it was less marked, for example, using 300 µg ml$^{-1}$ CP94, 130% and 80% increases of PpIX were obtained on exposure of explants to 1.2 and 2 mM ALA respectively (data not shown). Moreover, CP94 elevated human skin PpIX production in presence of 1.2 mM ALA-He by sixfold and 50% on exposure to 2 mM ALA-Me.

Controls incubated in the presence of CP94 without ALA exhibited fluorescence values equal to those obtained in the absence of the iron chelator. CP94 addition to cultures did not elevate the percentage of release of hydrophilic porphyrins into the medium in any of the conditions studied.

### Fluorescence microscopy

Figure 6 shows CCD images of fluorescence in human skin explants after 19 h incubation with the hexyl ester derivative (ALA-He) at 1.2 mM with 0.6 mM CP94 and, for comparison, 1.2 mM ALA alone. In all the sections examined the fluorescence is highest in the epidermis. In rat skin a similar biodistribution was observed, although fluorescence associated with hair follicles was more evident than in the human samples. Autofluorescence was only detectable in the stratum corneum.

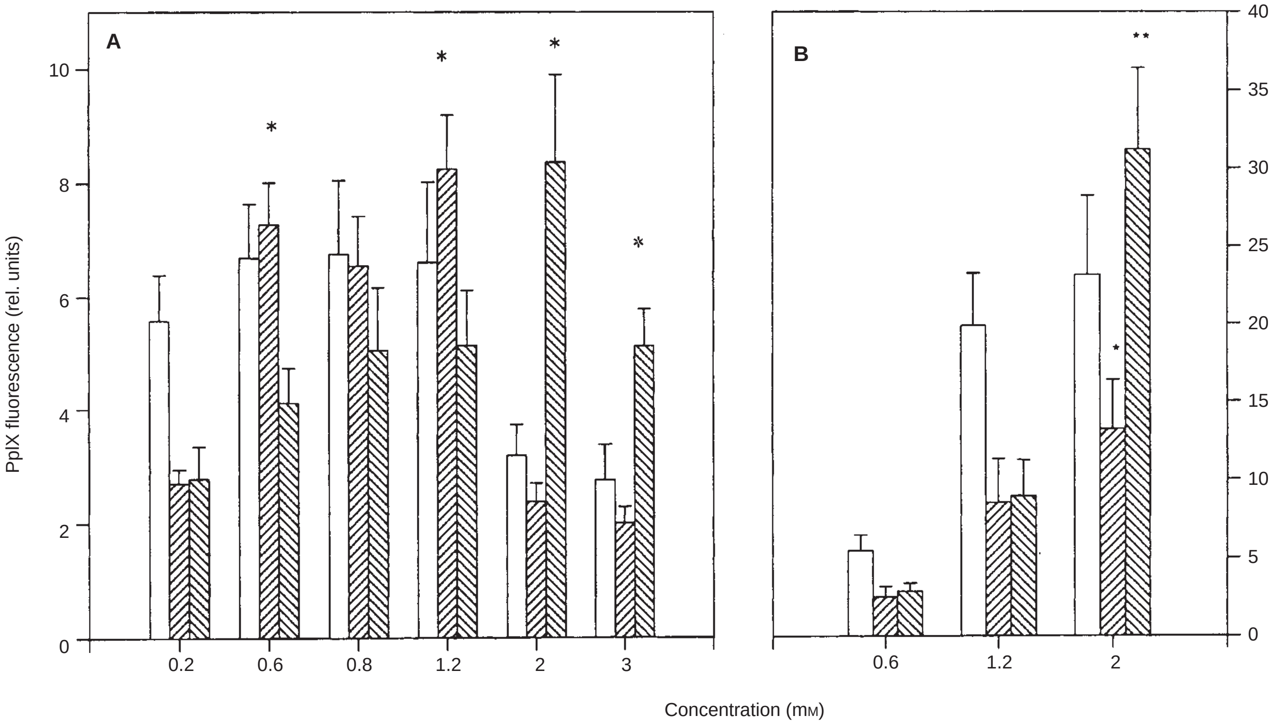

**Figure 4** PpIX fluorescence after 19 h exposure of rat (**A**) and human skin (**B**) to different concentrations of ALA (□), ALA-He (▨) and ALA-Me (▧). Direct skin fluorescence was measured at 410 nm excitation and 635 nm emission, and autofluorescence of explants incubated without ALA was subtracted. The bars show the means of four independent experiments performed in duplicate. Error bars show standard deviations. One asterisk indicates $P < 0.01$ and two, $P < 0.001$ compared to the corresponding ALA concentration

**Table 1** PpIX induced by ALA derivatives

| | | Rat | Human |
|---|---|---|---|
| ALA | | | |
| | 0.6 mM | 5.67 ± 0.43 | 5.83 ± 0.98 |
| | 1.2 mM | 6.80 ± 1.31 | 16.87 ± 3.45 |
| | 2 mM | 3.25 ± 0.48 | 21.15 ± 5.40 |
| ALA 3 | | | |
| | 0.6 mM | 0.35 ± 0.52 | 0.09 ± 0.11 |
| | 1.2 mM | 1.09 ± 0.09 | 0.15 ± 0.11 |
| | 2 mM | 1.00 ± 0.60 | 0.41 ± 0.24 |
| ALA 4 | | | |
| | 0.6 mM | 2.25 ± 1.31 | 0.03 ± 0.08 |
| | 1.2 mM | 1.40 ± 0.90 | 0.01 ± 0.20 |
| | 2 mM | 4.38 ± 0.01 | 0.39 ± 0.16 |
| ALA 5 | | | |
| | 0.6 mM | 1.18 ± 0.78 | 0.22 ± 0.04 |
| | 1.2 mM | 2.40 ± 0.82 | 0.24 ± 0.09 |
| | 2 mM | 3.23 ± 1.53 | 0.41 ± 0.22 |
| ALA 6 | | | |
| | 0.6 mM | 7.81 ± 0.95 | 2.87 ± 0.83 |
| | 1.2 mM | 7.33 ± 0.95 | 8.60 ± 1.59 |
| | 2 mM | 6.57 ± 3.12 | 29.60 ± 5.26 |
| ALA 7 | | | |
| | 0.6 mM | 1.75 ± 0.86 | 0.67 ± 0.25 |
| | 1.2 mM | 4.81 ± 0.76 | 2.83 ± 0.93 |
| | 2 mM | 5.56 ± 1.25 | 10.36 ± 2.28 |
| ALA 9 | | | |
| | 0.6 mM | 0.51 ± 0.09 | 0.08 ± 0.22 |
| | 1.2 mM | 1.92 ± 0.65 | 0.06 ± 0.26 |
| | 2 mM | 2.91 ± 0.78 | 0.24 ± 0.07 |
| ALA 10 | | | |
| | 0.6 mM | 0.96 ± 0.12 | 0.02 ± 0.09 |
| | 1.2 mM | 2.87 ± 2.19 | 0.16 ± 0.09 |
| | 2 mM | 1.43 ± 0.58 | 0.21 ± 0.39 |

Rat and human skin explants were incubated for 19 h in the presence of 0.6, 1.2 and 2 mM of ALA and ALA derivatives. In order to compare data, ALA was dissolved in DMSO in this experiment. PpIX fluorescence is expressed as arbitrary units measured at 410 nm excitation and 635 nm emission, and subtracting autofluorescence of explants from the same tissue sample incubated without ALA. Averages ± s.d. of three independent experiments performed in duplicate are shown.

### DISCUSSION

As can be seen from emission spectra of extracted porphyrins, the fluorescent species present in the samples was consistent with PpIX. Uroporphyrin, coproporphyrin and the various carboxylated intermediates are released into the medium by skin explants when incubated without serum (Fukuda et al, 1989). Under our conditions, no PpIX was fluorimetrically distinguished in the incubation media.

PpIX production as a function of ALA concentration was linear, which is consistent with earlier observations on mouse skin explants (Fukuda et al, 1989). The time-course of PpIX induction in the case of the methyl or hexyl esters may be affected by difference in diffusion through the dermis by the more hydrophobic compounds and may account for the more homogeneous distribution observed. The concentration dependence (Figure 4) may reflect the efficiency of conversion of the precursors to ALA by tissue esterases so that higher concentrations are required to release equivalent amounts of ALA. The greater effect on PpIX fluorescence in human skin explants of the methyl ester at high concentration may be due to a diminished rate of degradation. Topical administration of ALA esters (Peng et al, 1995, 1996) leads to a higher PpIX yield than that produced by the same dose of free ALA, but the factors involved are unclear.

In our work we found that higher ALA-Me concentrations are required to achieve the PpIX levels obtained by free ALA. These results agree with those of Washbrook and Riley (1997), who found that in hepatocyte cultures exposed to equimolar concentrations of ALA and ALA-Me, the latter was less effective at inducing the synthesis of PpIX, and are also in accordance with the results of Gaullier et al (1997). With the exception of the high-dosage methyl ester of ALA and compound 6, none of the derivatives examined were as active as ALA in inducing PpIX fluorescence. In the case of four of the derivatives no porphyrin production was observed. This may have been the result of some unrelated toxic action, perhaps due to its hydrophobic release of

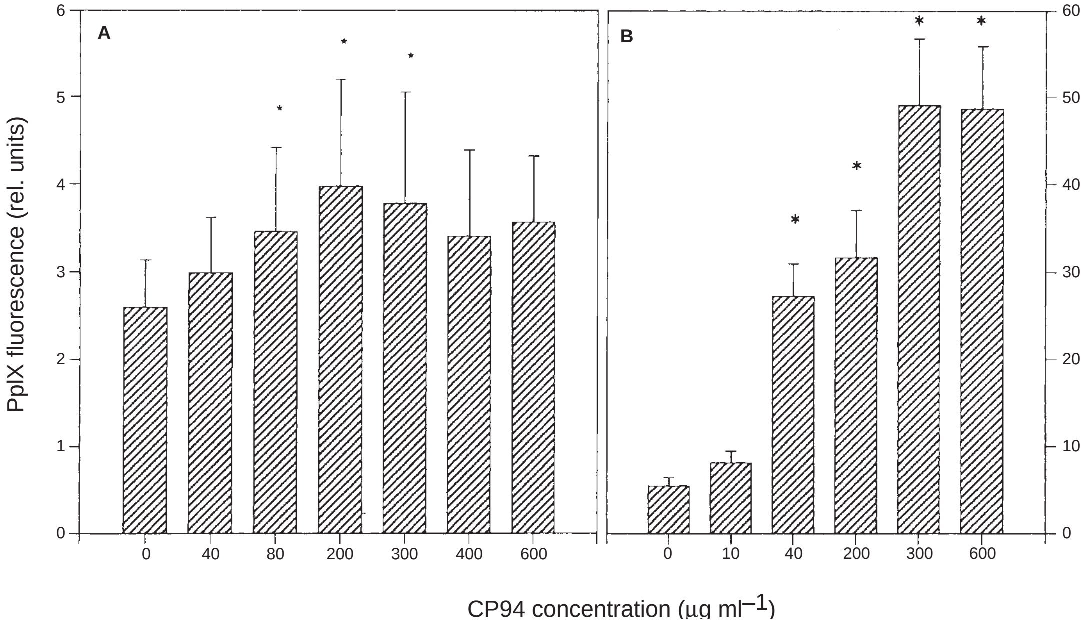

**Figure 5** PpIX fluorescence after 2 h exposure of rat (**A**) and human (**B**) skin to 0.6 mM ALA in the presence of different amounts of CP94 in the incubation media. Direct skin fluorescence was measured at 410 nm excitation and 635 nm emission, and autofluorescence of explants incubated without ALA was subtracted. The bars show the means of four independent experiments performed in duplicate. Error bars show standard deviations. Asterisks indicate $P < 0.05$

© 1999 Cancer Research Campaign

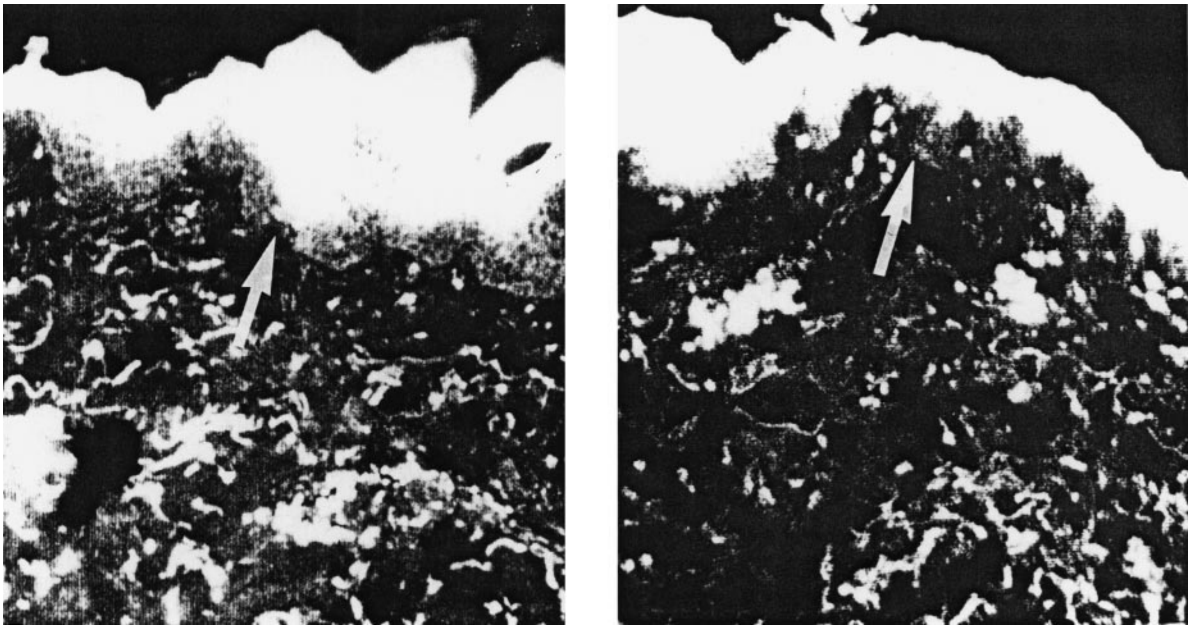

**Figure 6** Fluorescence micrographs (10 × objective, scale 500 × 550 micron) recorded with a CCD camera of human skin explants after 19 h incubation with (left) 1.2 mM ALA-He and 0.6 mM CP94, and (right) 1.2 mM ALA. In each case fluorescence is highest in the epidermis (see arrow)

short-chain carboxylic acids or the inability of the enzymes in the tissue to liberate the ALA from the pro-drug as in the case of the Mannich complexes or difficulties in their actual uptake.

In the remaining cases the efficiency of PpIX induction appears to differ between rat and human skin. In part the different thickness of the dermis may account for differences in diffusion, although in some instances (e.g. compounds 9 and 10) these are likely to be small. Modifications of stability of the ALA precursors should also be taken into account although the complexes are likely to be more susceptible to degradation than ALA itself. Nevertheless, the major factor would appear to be the effectiveness and tissue distribution of the ALA uptake and release mechanisms.

Because the initial step in the PpIX synthesis from ALA or its derivatives is their penetration through the plasma membrane, as indicated above, another very important parameter to be considered is the transmembrane transport of ALA and the extent to which the derivatives are capable of being translocated into the cytosol. This could be the rate-limiting step since most hydrolytic enzymes are located intracellularly. It is likely that the uptake of ALA and ALA derivatives might proceed by different mechanisms. The mechanism of ALA transport in human cells is not yet clear, but it has been well characterized in yeast (Bermudez Moretti et al, 1995). It would therefore be of interest to know whether ALA derivatives diffuse passively across the plasma membrane or have other active transport mechanisms similar or different from ALA.

At present it is not clear which enzymes might be involved in the liberation of ALA although in the case of compounds 3 and 4 and the methyl and hexyl esters it is likely that non-specific esterases are implicated and, in the case of compounds 5, 6 and 7 it may be that proteases are responsible for the release.

It is worthy of note that the explant culture system has been a useful and simple tool for testing ex-vivo the ability of organs to synthesize PpIX from ALA (Fukuda et al, 1989; Fritsch et al, 1997). Using floating explant cultures, the drugs are absorbed through the dermis to reach the epidermis, resembling the systemic pathway of drug delivery.

It is known that PpIX accumulation is enhanced by inhibition of ferrochelatase (Van Hilligersberg et al, 1992). CP94 is a powerful iron chelator which diminishes incorporation of iron into PpIX to form haem and may also inhibit the chelatase. Bech et al (1997) have shown in a cell culture system that CP94 markedly increases the yield of PpIX in cells exposed to ALA in the case of low ALA concentrations. In the experiments reported here we show that CP94 has a similar action in the case of human skin explants. Thus, the addition of CP94 may prove a useful method of increasing the sensitivity of PpIX bioassays as well as having possible clinical implications, as indicated by the results of Curnow et al (1998).

### ACKNOWLEDGEMENTS

A Casas is a Fellow of the Argentine National Research Council (CONICET) and A Batlle holds the post of Senior Research Scientist at the CONICET. We are grateful to the Association for International Cancer Research (AICR) for financial support. We thank Professor R Hider of King’s College London for supplies of CP94.

### REFERENCES

Bech O, Phillips D, Moan J and MacRobert A (1997) A hydroxypyridinone (CP94) enhances protoporphyrin IX formation in 5-aminolaevulinic acid-treated cells. *J Photochem Photobiol B: Biol* **41**: 136–144

Bedwell J, MacRobert A and Bown S (1992) Biodistribution of protoporphyrin IX in rat urinary bladder after intravesical instillation of 5-aminolaevulinic acid-induced PpIX in the DMH rat colonic tumour model. *Br J Cancer* **65**: 818–824

Berg K, Anholt H, Bech O and Mann J (1996) The influence of iron chelators on the accumulation of protoporphyrin IX in 5-aminolevulinic acid-treated cells. *Br J Cancer* **74**: 688–697

Bergmann M and Zervas L (1932) Über ein allgemeines Verfahren der Peptid-Synthese. *Chem Ber* **65**: 1192–1201

*© 1999 Cancer Research Campaign*

Bermúdez Moretti M, Correa García S, Chianellu M, Ramos E, Mattoon J and Batlle A (1995) Evidence that 4-aminobutyric acid and 5-aminolevulinic acid share a common transport system in *Saccharomyces cerevisiae*. *Int J Biochem Cell Biol* **27**: 169–173

Chang S, MacRobert A, Porter J and Bown S (1997) The efficacy of an iron chelator (CP94) in increasing cellular protoporphyrin IX following intravesical aminolaevulinic acid administration: an in vivo study. *J Photochem Photobiol B: Biol* **38**: 114–122

Correa García S, Bermúdez Moretti M, Rouvier Garay V and Batlle A (1998) Aminolevulinic acid transport through the blood–brain barrier. *Gen Pharmacol* **31**: 579–582

Curnow A, McIlroy B, Postle-Hacon M, Porter J, MacRobert A and Bown S (1998) Enhancement of 5-aminolaevulinic acid induced photodynamic therapy in normal rat colon using hydroxypyridinone iron chelating agents. *Br J Cancer* **78**: 1278–1282

Dietel W, Bolsen K, Dickson E, Fritsch C, Pottier R and Wendenburg R (1996) Formation of water-soluble porphyrins and protoporphyrin IX in 5-aminolevulinic-acid-incubated carcinoma cells. *J Photochem Photobiol B* **33**: 225–231

Fritsch C, Batz J, Bolsen K, Schutle K, Zumdick M, Ruzicka T and Goertz G (1997) Ex vivo application of 5-aminolevulinic acid induces high and specific porphyrin levels in human skin tumors: possible basis for selective photodynamic therapy. *Photochem Photobiol* **66**: 114–118

Fukuda H, Paredes S and Batlle A (1989) Tumor-localizing properties of porphyrins. In vitro studies using the porphyrin precursor, aminolevulinic acid, in free and liposome-encapsulated forms. *Drug Design Delivery* **5**: 133–139

Fukuda H, Paredes S and Batlle A (1992) Tumour-localizing properties of porphyrins. In vivo studies using free and liposome encapsulated aminolevulinic acid. *Comp Biochem Physiol* **102B**: 433–436

Gaullier JM, Berg K, Peng Q, Anholt H, Selbo PK, Ma LW and Moan J (1997) Use of 5-aminolevulinic acid esters to improve photodynamic therapy on cells in culture. *Cancer Res* **57**: 1481–1486

Hanania J and Malik Z (1992) The effect of EDTA and serum on endogenous porphyrin accumulation and photodynamic sensitization of human leukemic cells. *Cancer Lett* **65**: 127–131

Hider R, Kontoghiores G and Silver J (1982) Pharmaceutical Compositions, UK Patent GB 2118176A

Iida K, Takao Y, Ogai T and Kajiwara M (1998) Synthesis of delta-[N-IS] animolevulinic acid hydrochloride. *J Lab Comp Radiopharm* **39**: 797–802

Iinuma S, Farshi S, Ortel B and Hasan A (1994) A mechanistic study of cellular photodestruction with 5-aminolaevulinic acid-induced porphyrin. *Br J Cancer* **70**: 21–28

Kloek J and Beijersbergen van Henegouwen G (1996) Prodrugs for 5-aminolevulinic acid for photodynamic therapy. *Photochem Photobiol* **64**: 994–1000

Kloek J, Akkermans W and Beijersbergen van Henegouwen G (1998) Derivatives of 5-aminolevulinic acid for photodynamic therapy: enzymatic conversion into protoporphyrin. *Photochem Photobiol* **67**: 150–154

Navone N, Polo C, Frisardi A, Andrade N and Batlle A (1990) Heme biosynthesis in human breast cancer. Mimetic in vitro studies and some enzyme activity levels. *Int J Biochem* **22**: 1407–1411

Ortel B, Tanew A and Honigsmann H (1993) Lethal photosensitization by endogenous porphyrins of PAM cells: modification by desferrioxamine. *J Photochem Photobiol B* **17**: 273–278

Peng Q, Warloe T, Moan J, Heyerdahl H, Steen H, Giercksky K and Nesland J (1995) ALA derivative-induced protoporphyrin IX build-up and distribution in human nodular basal cell carcinoma. *Photochem Photobiol* 61 (abstract 82S)

Peng Q, Moan J, Warloe T, Iani V, Steen H, Bjørseth A and Nesland J (1996) Build-up of esterified aminolevulinic-acid-derivative-induced porphyrin fluorescence in normal mouse skin. *J Photochem Photobiol B: Biol* **34**: 95–96

Peng Q, Warloe T, Berg K, Moan J, Kongshang M, Giercksky K and Nesland J (1997) 5-Aminolevulinic acid-based photodynamic therapy: clinical research and future challenges. *Cancer* **79**: 2282–2308

Polo CF, Navone NM, Afonso SG, Vazquez ES, Buzaleh AM, Buanchi A, Schoua E and Batlle A (1988) Induction of porphyrin biosynthesis in tissue explants and the effect of anitmitotics. *ATLA* **16**: 137–147

Rebeiz N, Rebeiz C and Arkins C (1992) Photodestruction in tumor cells by induction of endogenous accumulation of protoporphyrin IX. Enhancement by 1,10-phenantroline. *Photochem Photobiol* **55**: 431–435

Takeya H (1992) Preparation of 5-aminolevulinic acid alkyl esters as herbicides. *Chem Abs* **116**: 189633 m

Tanaka T, Hotsuta Y and Takeya H (1992) Plant growth regulators containing 5-aminolevulinic acids and 5-alkanoylaminolevulinic acids. *Chem Abs* **122**: 308750 h

Van Hilligersberg R, Van der Berg J, Kort W, Terpstra O and Wilson J (1992) Selective accumulation of endogenously produced porphyrins in a liver metastasis model in rats. *Gastroenterology* **103**: 647–651

Walter G and Shalygo NV (1997) Location and fate of protoporphyrin IX accumulated in etiolated leaves and roots of *Zea mays* L. and *Pisum sativum* L. *J. Photochem Photobiol B: Biol* **40**: 175–182

Washbrook R and Riley PA (1997) Comparison of 5-aminolaevulinic acid and its methyl ester as an inducer of porphyrin synthesis in cultured cells. *Br J Cancer* **75**: 1417–1420

Washbrook R, Fukuda H, Batlle A and Riley PA (1997) Stimulation of tetrapyrrole synthesis in mammalian epithelial cells in culture by exposure to aminolaevulinic acid. *Br J Cancer* **75**: 381–387

 *© 1999 Cancer Research Campaign*
